# Evaluation of Hemodynamic Changes in Fetuses With Isolated Mild‐to‐Moderate Ventriculomegaly by Transabdominal Ultrasound

**DOI:** 10.1002/jum.15121

**Published:** 2019-08-25

**Authors:** Lijuan Sun, Lina Zhang, Na Zhang, Jijing Han, Zhen Li, Tiejuan Zhang, Ling Yao, Yuqing Ma, Li Wang, Yan Liu, Cuixia Guo, Qingqing Wu

**Affiliations:** ^1^ Department of Ultrasound, Beijing Obstetrics and Gynecology Hospital Capital Medical University Beijing China; ^2^ Department of Obstetrics, Beijing Obstetrics and Gynecology Hospital Capital Medical University Beijing China

**Keywords:** Doppler ultrasound, fetal cardiac function, modified myocardial performance index, prenatal diagnosis, ventriculomegaly

## Abstract

**Objectives:**

To investigate fetal hemodynamic alterations using transabdominal ultrasound in fetuses with isolated mild‐to‐moderate ventriculomegaly (VM).

**Methods:**

Fetuses diagnosed with isolated mild‐to‐moderate VM by transabdominal ultrasound were evaluated for hemodynamic changes, including changes in fetal cardiac function, the umbilical artery, the ductus venosus, and the middle cerebral artery. The fetuses with isolated mild‐to‐moderate VM were divided into 2 groups, namely, before 32 weeks’ gestation (20 weeks–31 weeks 6 days) and after 32 weeks’ gestation (32–38 weeks), and matched to corresponding healthy control fetuses.

**Results:**

The 53 fetuses with VM before 32 weeks had a longer mean isovolumetric relaxation time (IRT; mean ± SD, 42.9 ± 6.8 versus 40.4 ± 5.0 milliseconds; *P* < .05) and an apparently higher modified myocardial performance index 0.46 ± 0.06 versus 0.43 ± 0.05; *P* < .01) than the healthy control fetuses. The 43 fetuses with VM after 32 weeks had a significantly longer mean IRT (45.5 ± 6.7 versus 40.9 ± 7.2 milliseconds; *P* < .01) and a lower UA pulsatility index (0.81 ± 0.13 versus 0.89 ± 0.11; *P* < .01). The optimal cutoff levels for the IRT in the prediction of adverse perinatal outcomes were 40 and 43 milliseconds before and after 32 weeks, respectively (sensitivity, 100% versus 100%; specificity, 40.4% versus 50.0%; area under the curve, 0.601 versus 0.748; 95% confidence interval, 0.457–0.733 versus 0.590–0.869; *P* = .291 versus .005).

**Conclusions:**

Some fetuses with isolated mild‐to‐moderate VM may have impaired cardiac function, characterized by a higher modified myocardial performance index or longer IRT. This finding might be useful for improving fetal surveillance.

**Abbreviations**
DV: ductus venosus
ET: ejection time
ICT: isovolumetric contraction time
IRT: isovolumetric relaxation time
MCA: middle cerebral artery
Mod‐MPI: modified myocardial performance index
MPI: myocardial performance index
MRI: magnetic resonance imaging
PI: pulsatility index
PSV: peak systolic velocity
UA: umbilical artery
US: ultrasound
VM: ventriculomegaly

Enlargement of the atrium of the lateral ventricle during the development of the fetal brain is known as ventriculomegaly (VM). Fetal VM can be diagnosed by ultrasound (US) or magnetic resonance imaging (MRI) when the width of the unilateral or bilateral ventricles is greater than 10 mm. The Society for Maternal‐Fetal Medicine has recommended that VM be classified into 3 groups on the basis of prognosis: mild (10–12 mm), moderate (13–15 mm), and severe (>15 mm).^1^ Fetuses with severe VM often have a worse prognosis, with neurodevelopmental delays,^2^ and the parents of these fetuses usually elect to terminate the pregnancy. Most of fetuses with isolated mild or moderate VM have good outcomes, and the likelihoods of survival with normal neurodevelopment are greater than 90%^3^, ^4^, ^5^ for mild VM and 75% to 93% for moderate VM.^4^, ^6^, ^7^ However, isolated mild or moderate VM can still be associated with neurocognitive disorders and outcomes ranging from normal to severe damage. It has been reported that 15.7% of mild‐to‐moderate VM cases during pregnancy may have progression, and 16.7% of cases with progression show neurodevelopmental delays.^3^, ^8^ Fetal mild‐to‐moderate VM is still a challenging problem^8^ because providing parents with appropriate counseling and an accurate diagnosis is difficult.

As an indirect result of the 2‐child policy in China, the incidence of abnormalities in the fetal central nervous system is increasing. The prevalence of VM as a type of central nervous system anomaly is reported to range from 0.3 to 10 per 1000 births.^9^ It has been proven that once fetal mild or moderate VM is detected, monitoring the width of the lateral ventricle by US becomes essential because of its close association with the fetal prognosis.^3^ Nevertheless, other than routine measurements, no monitoring indicator for fetuses with VM has been described in the literature.

Cerebrospinal fluid is produced by the choroid plexus, exits the brain, and is ultimately absorbed through the venous system of the brain back into the circulation. Once “obstructive” VM occurs, there is a reduction in cerebral blood flow, or the dilated ventricle that may disrupt the balance and influence the general cardiovascular supply. The aim of this study was to investigate fetal hemodynamic alterations using transabdominal US, including changes in fetal cardiac function, the umbilical artery (UA), the ductus venosus (DV), and the middle cerebral artery (MCA), in fetuses with isolated mild‐to‐moderate VM.

## Materials and Methods

### *Patients*

This prospective cross‐sectional study was approved by the Institutional Review Board of Beijing Obstetrics and Gynecology Hospital, Capital Medical University (2016‐KY‐006‐02). Written informed consent to participate in the study was obtained from each patient.

The inclusion criteria for the research groups were as follows: fetuses with a diagnosis of isolated mild‐to‐moderate VM at 20 to 38 weeks’ gestation by transabdominal US between July 2016 and July 2018 in the Department of Ultrasound and the Prenatal Diagnosis Center of Beijing Obstetrics and Gynecology Hospital. Mild‐to‐moderate VM was defined as a lateral ventricle with a width of 10 to 15 mm according to the Society for Maternal‐Fetal Medicine.^1^ Fetal isolated VM was considered when there was no evidence showing other structural anomalies or chromosomal abnormalities at the time of presentation.^5^ Prenatal MRI was recommended to the parents to exclude other fetal central nervous system anomalies, such as cortical anomalies, agenesis of the corpus callosum, and posterior fossa malformations. Amniocentesis and a chromosomal microarray analysis were recommended for the pregnant women to determine the fetal karyotype and copy number variations to rule out fetal chromosomal abnormalities. The exclusion criteria were as follows: multiple pregnancies, VM associated with chromosomal anomalies or other congenital malformations, fetal arrhythmia, fetal growth restriction, obstetric complications, alcohol addiction or smoking, and drug use.

Based on differences in Doppler US indicators with increasing gestational age, the fetuses with isolated mild‐to‐moderate VM were divided into 2 groups, namely, fetuses before 32 weeks’ gestation (20 weeks–31 weeks 6 days) and fetuses after 32 weeks’ gestation (32–38 weeks) and were matched with the same sample of singleton pregnancies with normal maternal and fetal conditions, respectively. The pregnant women had no clinically relevant gynecologic problems, obstetric problems, or medical histories and had completely normal pregnancy follow‐up assessments. The maternal age and gestational age at the time of the US evaluation were matched between the study and control groups.

### *Ultrasound Examination*

A fetal US examination was conducted for each fetus with an WS80A US system equipped with a transabdominal transducer of 1 to 7 MHz (Samsung, Medison, Korea). Obstetric conventional US examinations and Doppler evaluations were performed as described by the International Society of Ultrasound in Obstetrics and Gynecology practice guidelines.^10^, ^11^, ^12^

#### *Obstetric Conventional US Examination*

**Figure 1:**
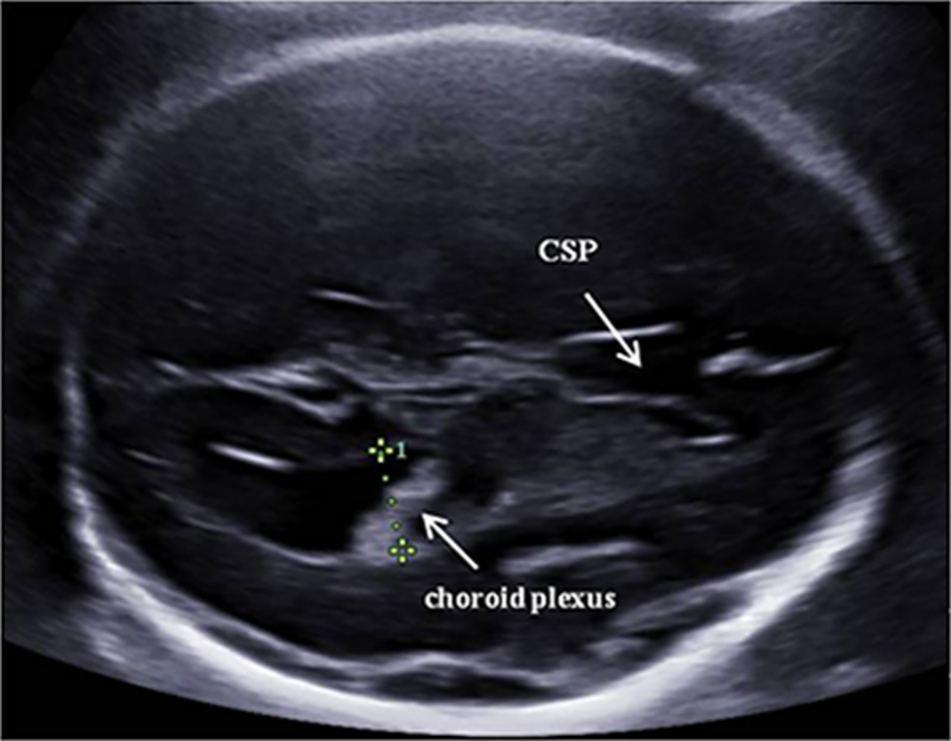
Measurement of the atrium of the lateral ventricles. The calipers were placed at the internal margin of the medial and lateral walls of the atria on an axis perpendicular to the long axis of the lateral ventricle. CSP indicates cavum septi pellucidi.

Fetal biometric parameters and the amount of amniotic fluid were measured according to the International Society of Ultrasound in Obstetrics and Gynecology practice guidelines.^12^ The measurement of the fetal lateral ventricle was performed with a view of the lateral ventricles full of echogenic choroid plexuses in the transverse plane of the fetal brain. The calipers were placed at the inner edges of the ventricle walls and aligned perpendicular to the ventricular cavity (Figure 1).^10^

#### *Doppler Evaluation (General Hemodynamic Assessment)*

**Figure 2:**
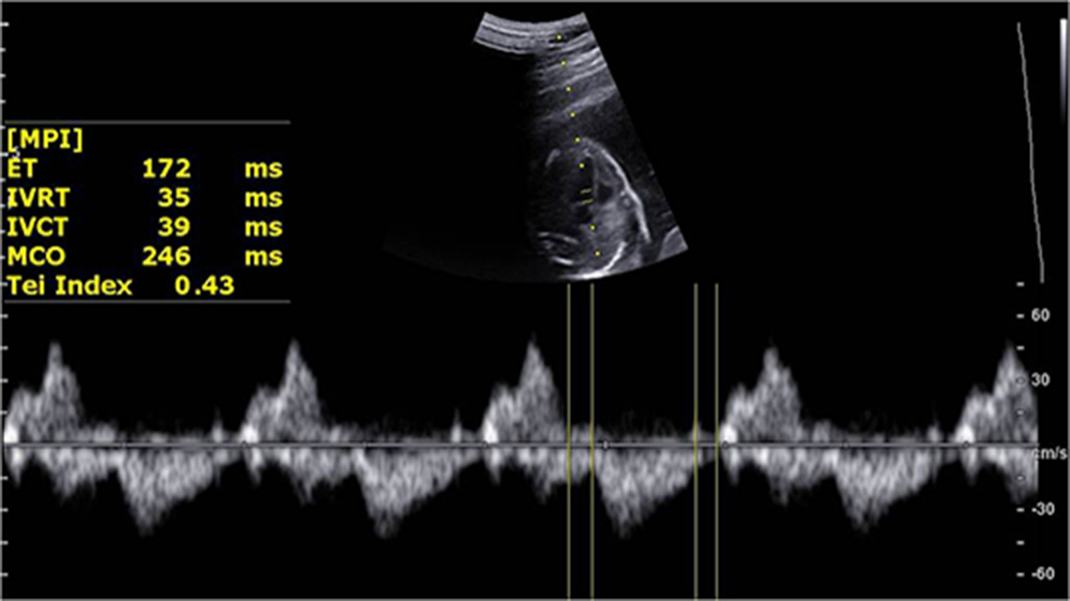
Normal Mod‐MPI (Tei index) Doppler image. The Doppler sample was located at the cross point between the inflow and outflow of the left ventricle, which included the leaflet of the mitral valve and aortic valve with a 3‐ to 4‐mm sample size. MCO, mitralvalve closing; and open time, ICT + IRT + ET.

*Fetal Cardiac Function*—The fetal left ventricular myocardial performance index (MPI) can evaluate global myocardial function as a noninvasive Doppler‐derived technique.^13^ A modified myocardial performance index (Mod‐MPI) was used, which showed smaller differences and higher interobserver and intraobserver agreement than the MPI in this study.^14^ The Mod‐MPI was measured when the opening and closure of the mitral valve and aortic valve in the transverse 4‐chamber image with an apical heart was clearly shown.^14^ The Doppler sample was located at the cross point between the inflow and outflow of the left ventricle, which included the leaflets of the mitral valve and aortic valve with a 3‐ to 4‐mm sample size. The insonation angle was less than 30°. The Doppler sweep velocity was set at 540 Hz; the wall motion filter was set at 100 Hz; and the scale was set at 55 cm/s. The isovolumetric contraction time (ICT), isovolumetric relaxation time (IRT), and ejection time (ET) were measured. The MPI was equal to (ICT + IRT)/ET (Figure 2). The fetal heart rate was also calculated.

**Figure 3:**
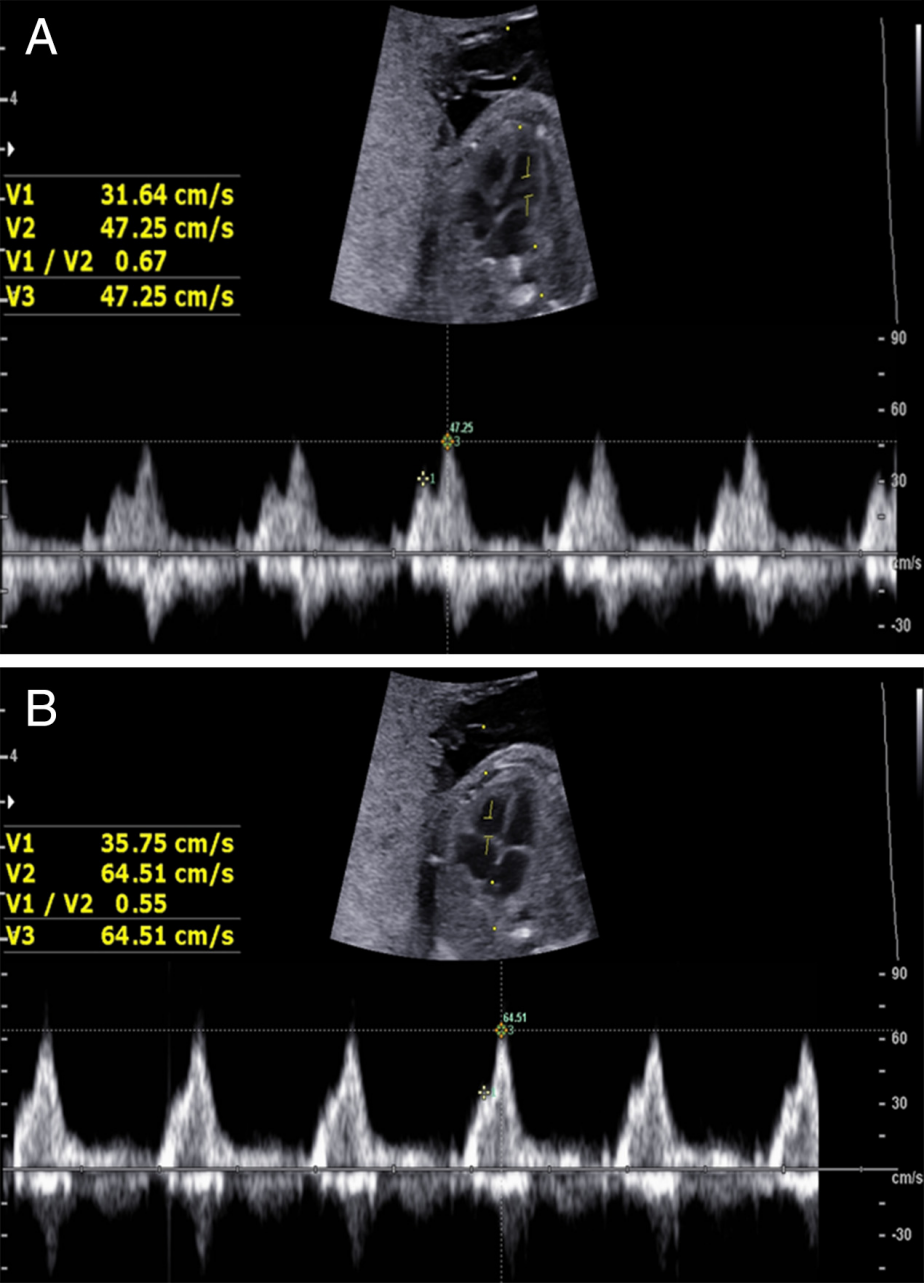
Measurement of the E wave (V1), A wave (V2), and E/A ratio from both the fetal mitral valve (**A**) and fetal tricuspid valve (**B**).

The E wave (early ventricular filling) peak velocity, A wave (active atrial filling) peak velocity, and E/A ratio from both the mitral valve and tricuspid valve can reflect fetal cardiac diastolic function. They were detected in the apical 4‐chamber view by the Doppler sample located at the tip of the atrioventricular valves during diastole (Figure 3).

**Figure 4:**
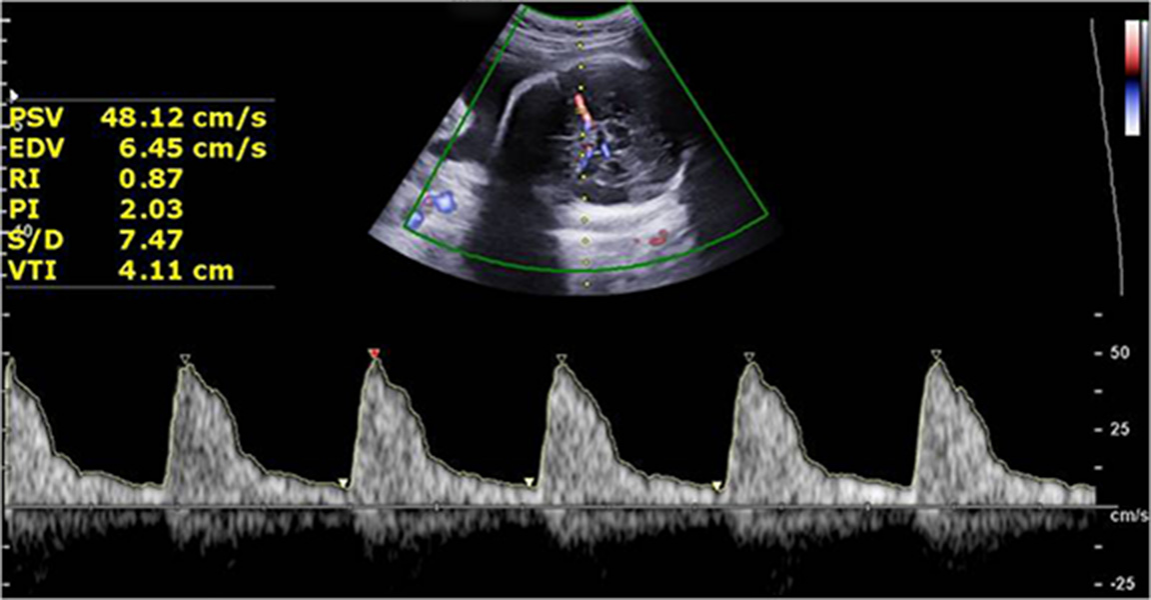
Doppler measurement of the MCA. EDV indicates end‐diastolic velocity; RI, resistive index; S/D, systolic‐to‐diastolic ratio; and VTI, velocity time integral.

*Fetal MCA*—Fetal MCA Doppler indicators, including the pulsatility index (PI) and peak systolic velocity (PSV), were measured. The Doppler sample was located near the origin of the MCA in the internal carotid artery, at the proximal third of the MCA (Figure 4). The insonation angle between the US beam and the direction of blood flow was kept as close as possible to 0°. No unnecessary pressure was placed on the fetal head.^11^

**Figure 5:**
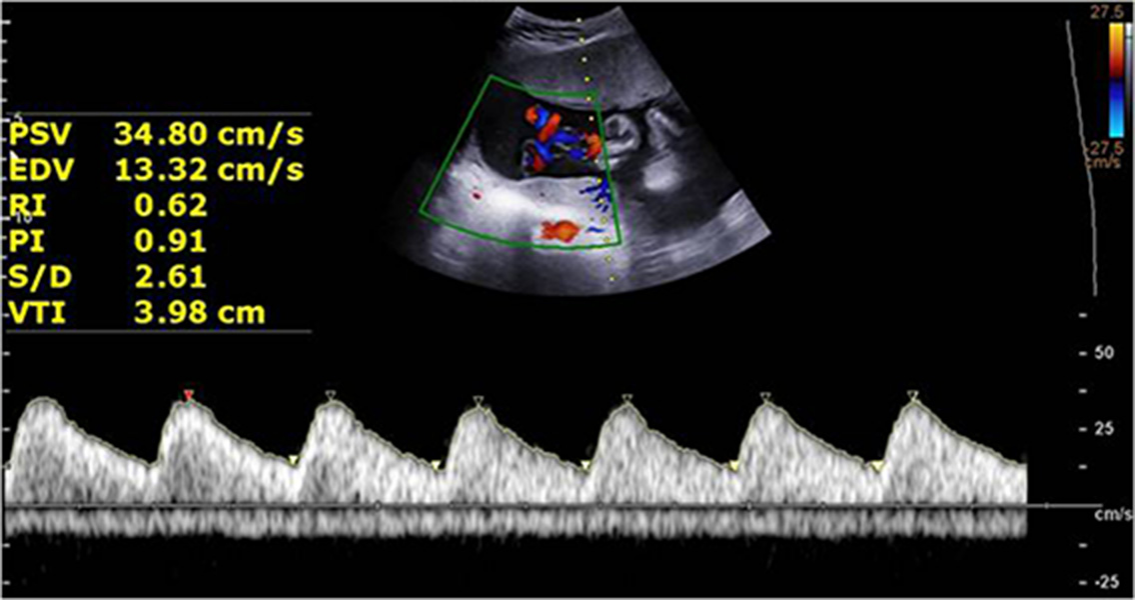
Doppler measurement of the UA. Abbreviations are as in Figure 4.

*Fetal UA*—The UA PI was measured in the free loop of the umbilical cord between the fetal insertion and placental insertion sites (Figure 5).

**Figure 6:**
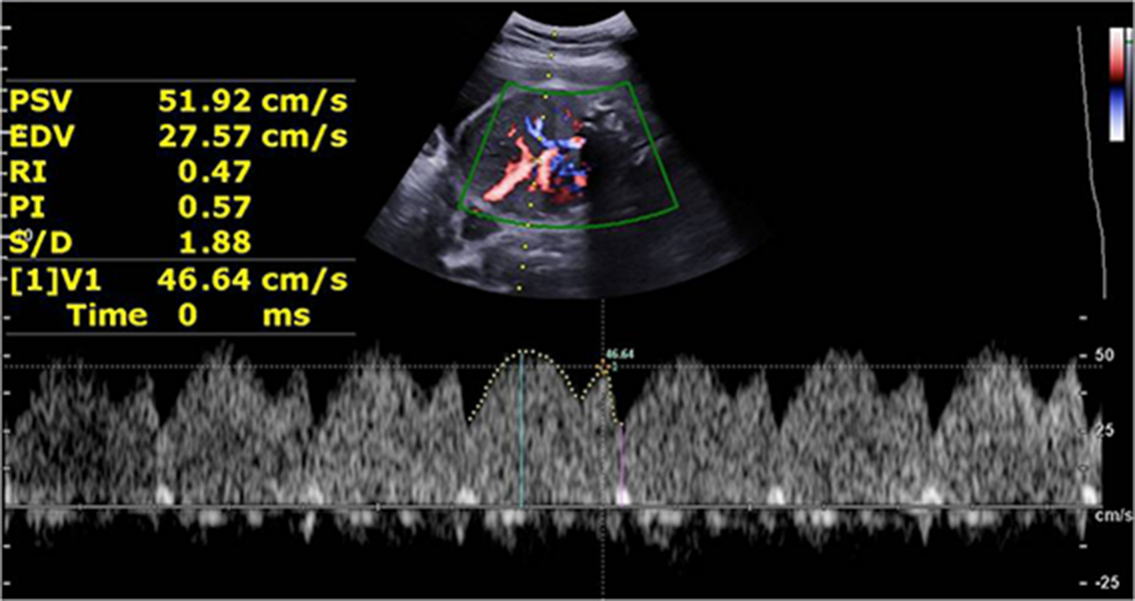
Doppler measurement of the DV. Abbreviations are as in Figure 4.

*Fetal DV*—The standard sampling site for the DV Doppler measurement was confirmed by color flow mapping showing high‐speed flow at the narrow entrance (Figure 6).^11^ The DV PI measurement was achieved in the sagittal plane or oblique section of the fetal trunk.

### *Follow‐up*

Pregnancy outcomes including gestational age at birth, birth weight, and Apgar score were followed and recorded. An adverse pregnancy outcome was considered when at least 1 of the following serious conditions occurred: nonreassuring fetal heart rate tracing, the presence of meconium in the amniotic fluid, neonatal intensive care unit admission, progression to severe VM, and neonatal intracranial hemorrhage.

### *Statistical Analysis*

Data were analyzed with SPSS version 23.0 software (IBM Corporation, Armonk, NY). All numeric data are expressed as mean ± standard deviation. Continuous variables between 2 groups were compared by the independent‐samples *t* test. A χ^2^ test was used to compare the rates of adverse pregnancy outcomes and the percentages of Apgar scores of less than 7 between the fetal isolated VM and control groups. Receiver operating characteristic curves were used to calculate the sensitivity and specificity of Doppler indicators for predicting adverse pregnancy outcomes. *P* < .05 was considered statistically significant.

## Results

Ninety‐five women with singleton pregnancies with fetal isolated mild‐to‐moderate VM were recruited into this study, including 53 fetuses (55.8%) before 32 weeks’ gestation and 42 cases (44.2%) after 32 weeks’ gestation. The mean gestational age was 29 (range, 20–38) weeks. The mean age of the pregnant women was 31 (range, 23–39) years. Seventy‐one pregnant women underwent prenatal MRI, and 15 cases were followed with brain MRI after birth among the fetuses who had no MRI prenatally. Amniocentesis was performed for 29 pregnant women, and 19 of those who declined underwent noninvasive prenatal testing for fetal aneuploidy.

The clinical characteristics of the study groups (fetuses with isolated mild‐to‐moderate VM) and the healthy control groups are shown in Table 1 (<32 weeks) and Table 2 (>32 weeks). There were no statistically significant differences in terms of maternal age, fetal US biometric measurements, or gestational age at the time of the US evaluation between the study groups and control groups.

**Table 1:** Clinical Characteristics of the Study Group and the Healthy Control Group Before 32 Weeks’ Gestation

Characteristic	Control Group (n = 53)	Study Group (n = 53)	*P*
Maternal age, y	30.6 ± 3.5	30.7 ± 3.7	.797
Gestational age at US, wk	27.9 ± 3.6	27.7 ± 2.6	.737
Biparietal diameter, cm	7.1 ± 1.1	7.0 ± 0.7	.478
Head circumference, cm	25.8 ± 3.5	25.8 ± 2.4	.997
Abdominal circumference, cm	23.5 ± 3.6	23.2 ± 2.7	.671
Femur length, cm	5.2 ± 0.8	5.1 ± 0.7	.427

**Table 2:** Clinical Characteristics of the Study Group and the Healthy Control Group After 32 Weeks’ Gestation

Characteristic	Control Group (n = 42)	Study Group (n = 42)	*P*
Maternal age, y	31.6 ± 3.9	31.4 ± 4.1	.732
Gestational age at US, wk	34.3 ± 1.5	34.4 ± 1.9	.718
Biparietal diameter, cm	8.6 ± 0.4	8.7 ± 0.5	.412
Head circumference, cm	30.9 ± 1.0	31.5 ± 1.6	.051
Abdominal circumference, cm	30.0 ± 1.7	30.2 ± 2.2	.765
Femur length, cm	6.6 ± 0.4	6.5 ± 0.4	.312

**Figure 7:**
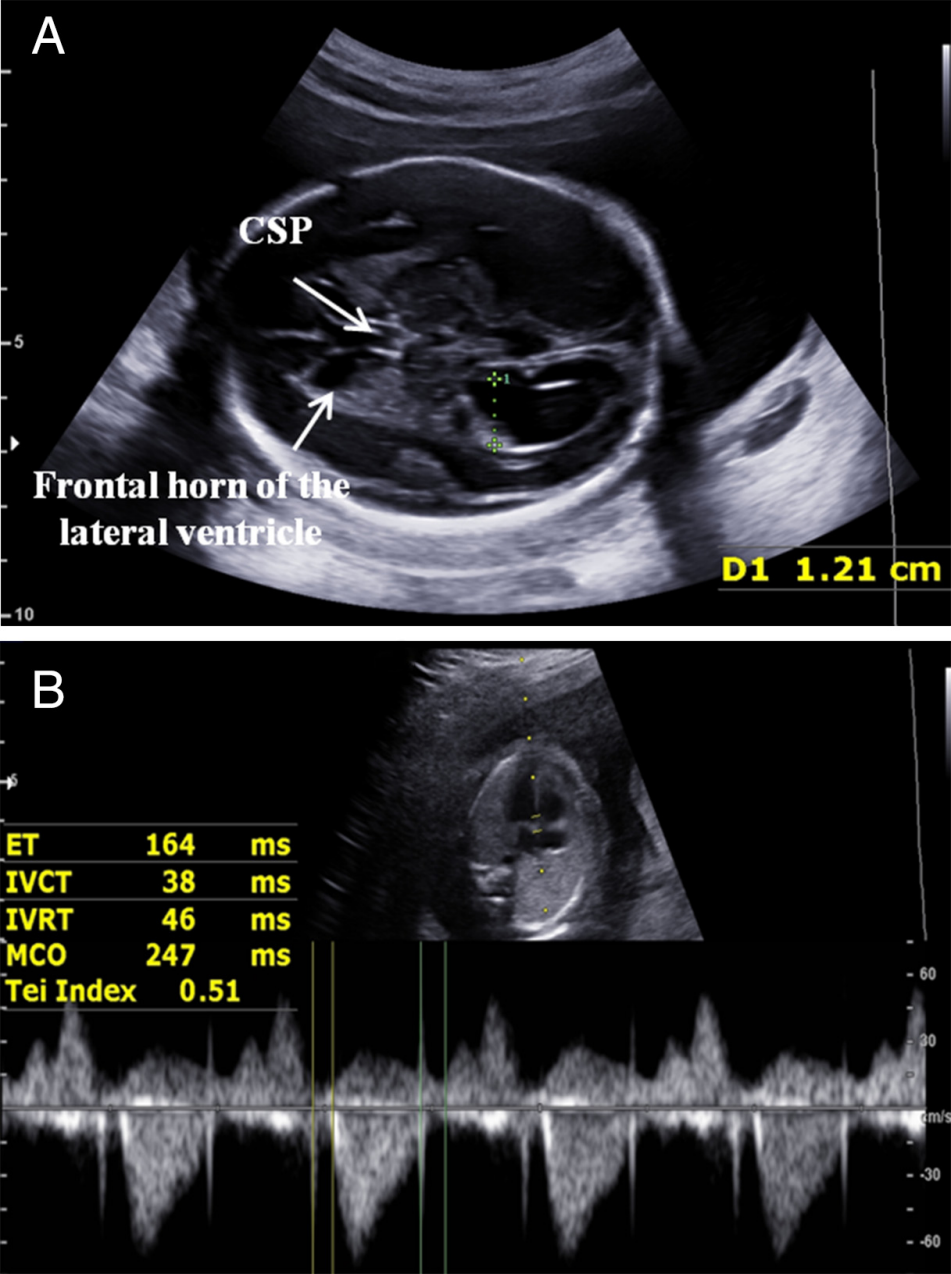
Image from a fetus at 23 weeks’ gestation with isolated moderate VM (**A**). The fetal Mod‐MPI (Tei index) was 0.51, and the IRT was 46 milliseconds, which showed that fetal cardiac function was decreased (**B**). CSP indicates cavum septi pellucidi; MCO, mitralvalve closing; and open time, ICT + IRT + ET.

The comparisons of the Doppler parameters and perinatal outcomes between the study groups and control groups are shown in Table 3 (<32 weeks) and Table 4 (>32 weeks). The fetal Mod‐MPI was significantly higher in the fetuses with isolated mild‐to‐moderate VM before 32 weeks’ gestation than in the control group (0.46 ± 0.06 versus 0.43 ± 0.05; *P* < .01; Figure 7). Both the ICT and ET showed that there were no statistically significant differences between the groups (both *P* > .05), whereas the IRT was longer in the study group than in the control group (42.9 ± 6.8 versus 40.4 ± 5.0 milliseconds; *P* < .05). Table 4 shows that the fetuses with isolated mild‐to‐moderate VM after 32 weeks’ gestation had a significantly longer IRT than the healthy control fetuses (45.5 ± 6.7 versus 40.9 ± 7.2 millisecond; *P* < .01); however, the fetal Mod‐MPI, ICT, and ET showed no statistically significant differences between the groups (all *P* > .05). The UA PI was also significantly lower in the study group than in the control group (0.81 ± 0.13 versus 0.89 ± 0.11; *P* < .01). The incidences of adverse perinatal outcomes in both study groups were higher (Tables 3 and 4; *P* < .05).

**Table 3:** Doppler Parameters and Perinatal Outcomes of the Study Group and the Healthy Control Group Before 32 Weeks’ Gestation

Parameter	Control Group (n = 53)	Study Group (n = 53)	*P*
Fetal heart rate, beats/min	147 ± 10	150 ± 9	.238
IRT, ms	40.4 ± 5.0	42.9 ± 6.8	.038
ICT, ms	32.4 ± 6.9	35.3 ± 9.4	.068
ET, ms	171.6 ± 11.5	170.5 ± 10.8	.615
Mod‐MPI, ms	0.43 ± 0.05	0.46 ± 0.06	.004
MV E velocity, cm/s	33.5 ± 7.6	32.9 ± 6.5	.646
MV A velocity, cm/s	51.1 ± 9.0	52.0 ± 7.5	.606
MV E/A ratio	0.65 ± 0.11	0.63 ± 0.09	.371
TV E velocity, cm/s	38.5 ± 8.2	38.8 ± 8.6	.850
TV A velocity, cm/s	57.0 ± 9.2	56.3 ± 8. 8	.719
TV E/A ratio	0.68 ± 0.11	0.68 ± 0.11	.945
MCA PI	1.87 ± 0.36	1.85 ± 0.38	.853
MCA PSV, cm/s	37.5 ± 10.8	36.0 ± 8.7	.446
UA PI	1.02 ± 0.18	1.02 ± 0.26	.849
DV PI	0.56 ± 0.17	0.57 ± 0.19	.917
Gestational age at birth, wk	39.2 ± 1.1	39.0 ± 1.7	.529
Birth weight, g	3405 ± 349	3455 ± 334	.537
Apgar score < 7, n (%)	1/53 (1.9)	2/53 (3.8)	>.999
Adverse perinatal outcome, n (%)	0/53	6/53 (11.3)	.027
Nonreassuring fetal heart rate tracing	0	0	
Presence of meconium in amniotic fluid	0	1/53 (1.9)	
NICU admission	0	0	
Progression to severe VM	0	5/53 (5.7)	
Intracranial hemorrhage	0	0	

MV indicates mitral valve; NICU, neonatal intensive care unit; and TV, tricuspid valve.

**Table 4:** Doppler Parameters in the Study Group and the Healthy Control Group After 32 Weeks’ Gestation

Parameter	Control Group (n = 42)	Study Group (n = 42)	*P*
Fetal heart rate, beats/min	144 ± 11	147 ± 11	.204
IRT, ms	40.9 ± 7.2	45.5 ± 6.7	.003
ICT, ms	33.9 ± 8.8	32.1 ± 8.8	.377
ET, ms	168.6 ± 14.1	168.5 ± 11.7	.957
Mod‐MPI, ms	0.44 ± 0.05	0.47 ± 0.07	.083
MV E velocity, cm/s	36.0 ± 7.6	39.0 ± 8.4	.085
MV A velocity, cm/s	52.5 ± 7.3	54.7 ± 8.9	.212
MV E/A ratio	0.68 ± 0.10	0.72 ± 0.17	.214
TV E velocity, cm/s	42.3 ± 9.2	47.3 ± 12.8	.046
TV A velocity, cm/s	60.0 ± 9.6	61.5 ± 11.4	.527
TV E/A ratio	0.70 ± 0.10	0.75 ± 0.13	.047
MCA PI	1.79 ± 0.30	1.73 ± 0.40	.482
MCA PSV, cm/s	50.0 ± 8.9	50.6 ± 11.8	.796
UA PI	0.89 ± 0.11	0.81 ± 0.13	.002
DV PI	0.51 ± 0.19	0.57 ± 0.25	.265
Gestational age at birth, wk	38.9 ± 0.9	38.9 ± 1.3	.899
Birth weight, g	3422 ± 431	3628 ± 363	.055
Apgar score < 7, n (%)	0/42	1/42 (2.4)	>.999
Adverse perinatal outcome, n (%)	0	8/42 (19.0)	<.001
Nonreassuring fetal heart rate tracing	0	4/42 (7.1)	
Presence of meconium in amniotic fluid	0	3/42 (7.1)	
NICU admission	0	1/42 (2.4)	
Progression to severe VM	0	1/42 (2.4)	
Intracranial hemorrhage	0	1/42 (2.4)	

Abbreviations are as in Table 3.

**Figure 8:**
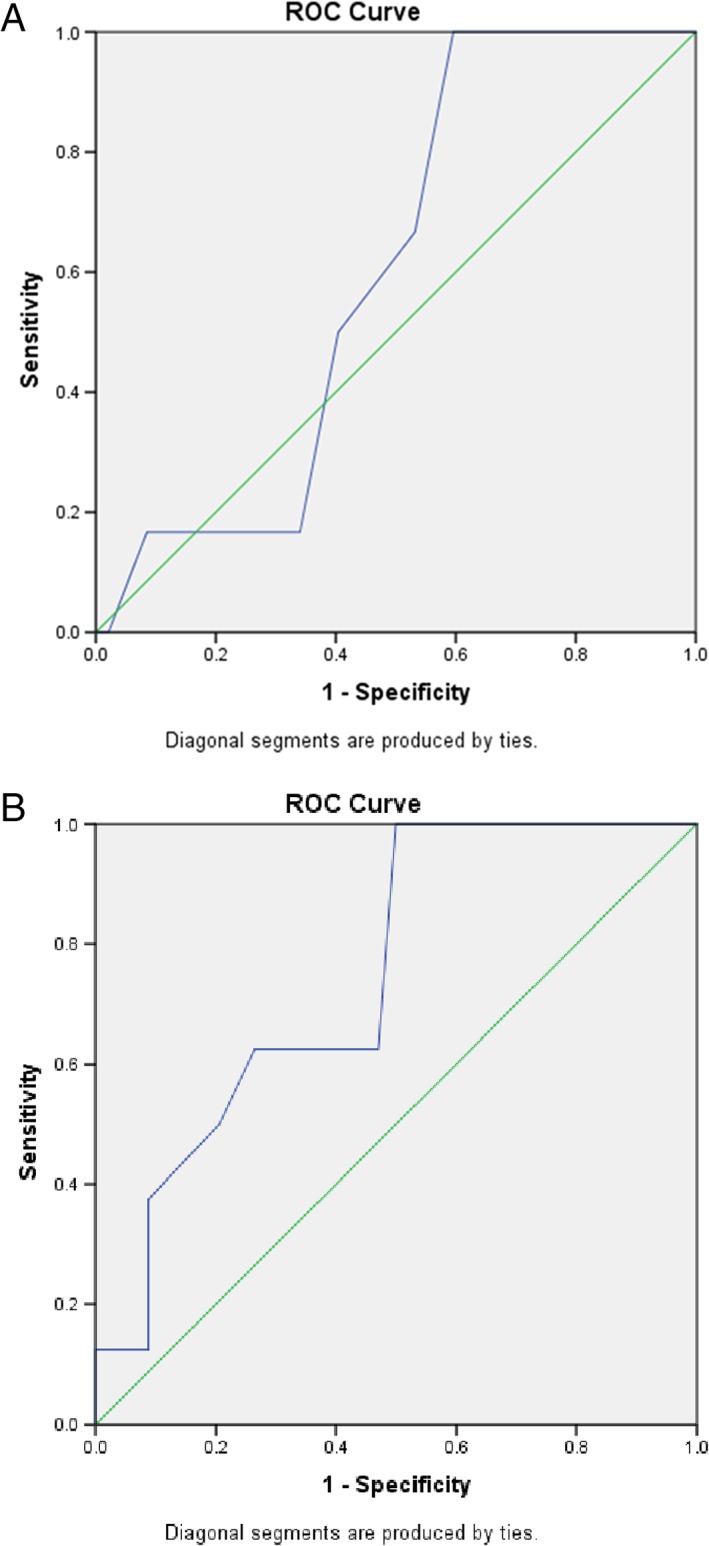
Receiver operating characteristic curves for the IRT in predicting adverse perinatal outcomes in the study groups before 32 weeks’ gestation (**A**) and after 32 weeks’ gestation (**B**).

The predictive performances of the IRT, Mod‐MPI, and UA PI for adverse perinatal outcomes are shown in Table 5 and Figure 8. The optimal cutoff levels for the IRT for the prediction of adverse perinatal outcomes, which were dependent on the Youden index, were greater than 40 and greater than 43 milliseconds before and after 32 weeks, respectively (sensitivity, 100% versus 100%; specificity, 40.4% versus 50.0%; area under the curve, 0.601 versus 0.748; 95% confidence interval, 0.590–0.869 versus 0.497–0.799; *P* = .291 versus .005). There were significant differences in the IRT value after 32 weeks (*P* = .048), whereas there were no significant differences in the IRT value before 32 weeks, Mod‐MPI, or UA PI (all *P* > .05) among the fetuses with isolated mild‐to‐moderate VM with or without adverse perinatal outcomes (Table 6).

**Table 5:** Predictive Performances of IRT, Mod‐MPI, and UA PI in Terms of Adverse Perinatal Outcomes

Parameter	AUC	95% CI	*P*	Youden Index	Sensitivity, %	Specificity, %
<32 wk						
IRT	0.601	0.457–0.733	0.291	0.4043	100	40.4
Mod‐MPI	0.535	0.393–0.674	0.779			
>32 wk						
IRT	0.748	0.590–0.869	0.005	0.5000	100%	50.0
UA PI	0.618	0.455–0.763	0.248			

AUC indicates area under the curve; and CI, confidence interval.

**Table 6:** Isovolumetric Relaxation Time, Mod‐MPI, and UA PI for Fetuses With Isolated Mild‐to‐Moderate VM With or Without Adverse Perinatal Outcomes

Parameter	VM Without Adverse Outcomes	VM With Adverse Outcomes	*P*
<32 wk			
IRT, ms	42.7 ± 7.0	44.5 ± 4.8	.544
Mod‐MPI, ms	0.46 ± 0.06	0.48 ± 0.07	.552
>32 wk			
IRT, ms	44.8 ± 6.3	50.6 ± 6.7	.048
UA PI	0.80 ± 0.14	0.87 ± 0.11	.213

## Discussion

Because fetuses with isolated mild or moderate VM often have a good prognosis, we would like these cases to be able to be evaluated comprehensively and thoroughly. This study investigated the effects of isolated mild‐to‐moderate VM on the fetal blood circulation and demonstrated that these fetuses had damaged heart function, characterized by a higher Mod‐MPI and longer IRT than the healthy control fetuses.

It is of critical importance for the correct assessment of fetal myocardial function while evaluating high‐risk fetuses. These evaluations may be helpful in timing the delivery appropriately and may be life saving for the fetus based on the early identification of slight alterations in fetal myocardial performance. Nevertheless, to the best of our knowledge, the assessment of fetal cardiac function is still evolving. The MPI has been proven to be independent of gestational age and the fetal heart rate, and the MPI can be used for the initial evaluation of global cardiac function in healthy fetuses and fetuses with congenital malformations.^15^ The IRT is the most important component of the MPI, and its derangement in the MPI indicates the earliest change in cardiac function impairment in diseased states.^16^ We found that fetuses with isolated mild‐to‐moderate VM before 32 weeks’ gestation had not only a significantly higher Mod‐MPI but also a longer IRT than the control group, whereas fetuses with isolated mild‐to‐moderate VM after 32 weeks’ gestation only had a significantly longer IRT, with no significant difference in the Mod‐MPI. There may be a compensatory reaction that occurs through the shortening of the ICT to keep the Mod‐MPI stabilized for fetuses with isolated mild‐to‐moderate VM after 32 weeks’ gestation.

We found that the IRT had an advantage in predicting the adverse perinatal outcomes of fetuses with isolated mild‐to‐moderate VM after weeks (area under the curve, 0.748; *P* < .05). At the optimal cutoff level for the IRT (>43 milliseconds >32 weeks), which was determined by the Youden index, the sensitivity reached 100%, although the specificity was low (50.0%). This finding suggested that adverse perinatal outcomes should be given attention when fetal isolated mild‐to‐moderate VM is associated with a longer IRT, which may be considered a screening Doppler indicator. Significant differences in the IRT values of the cases with and without adverse perinatal outcomes (*P* = .048) were not evident, which may have been because of the small sample size.

In recent years, several perinatal conditions have been shown to affect fetal cardiac function. For growth‐restricted fetuses and fetuses of mothers with preeclampsia, the leading cause of fetal cardiac dysfunction is increased placental vascular resistance, resulting in an increased cardiac afterload and a higher MPI.^17^, ^18^, ^19^ However, we considered that the pathogenesis of fetal cardiac dysfunction caused by VM may be different from those of fetal growth restriction and the mothers with preeclampsia because there was never an elevated UA PI in the study group. Instead, the fetuses with isolated mild‐to‐moderate VM after 32 weeks’ gestation had a significantly lower UA PI. The MPI was also applied to postdate pregnancies,^20^ twin‐twin transfusion syndrome,^21^ and fetuses in women with gestational impaired glucose tolerance^22^ by other authors. Nevertheless, few studies have focused on assessing cardiac function in fetuses with VM in the literature. A significantly decreasing cardiac output was observed in cases with chronic hydrocephalus caused by surgical induction in a dog model.^23^ This result implied that the chronic hydrocephalus in the animal model was associated with damaged cardiac function, which was similar to our observation. The reduction in local cerebral blood flow and a dilated ventricle occurring in cases with VM may influence the general cardiovascular supply. However, the pathogenesis of VM actions on fetal cardiac function is still not clear, and further studies are needed.

Interestingly, the fetuses with isolated mild‐to‐moderate VM after 32 weeks’ gestation had a significantly lower UA PI. This demonstrated that the cardiac afterload of fetuses was decreased, leading to an ICT shortening or an ET increase so that the Mod‐MPI remained normal with this prerequisite of a longer IRT. This phenomenon that occurred in fetuses with isolated mild‐to‐moderate VM after 32 weeks’ gestation was consistent with the hemodynamic changes and further explained why there was no statistically significant difference in the fetal Mod‐MPI between the study group after 32 weeks’ gestation and the control group. Although the mechanism underlying the fetal UA PI decrease was not clear, it further confirmed that the pathogenesis of VM actions on fetal cardiac function was different from those of fetal growth restriction and mothers with preeclampsia.

This study showed that there were no statistically significant differences in the MCA PI or MCA PSV between the study and control groups. This finding was similar to that in a study by Malinger et al,^24^ who demonstrated that unilateral or bilateral mild VM was not related to fetal MCA Doppler changes. However, a fetal MCA Doppler abnormality (absent or reversed blood flow during diastole) has been proven to be a poor prognostic sign, with obviously high perinatal mortality in cases with hydrocephalus.^25^ Our observation, in contrast to the finding of hydrocephalus that has been shown to be related to increased resistance to the flow velocity, is because we believe that mild‐to‐moderate VM has a different pathophysiologic process from severe VM or hydrocephalus, with increased local pressure or hemorrhagic events.^24^

There were several limitations in our study. First, our preliminary research included relatively few amniocentesis procedures, which may have failed to entirely rule out the possibility of fetal chromosomal abnormalities, so the results still need to be verified by a prospective cohort study with a larger sample size. Second, the level of technical consistency can be determined by analyzing the intraobserver and interobserver reliabilities of the Mod‐MPI. Finally, further study with long‐term follow‐up for cardiac conditions, including assessments of the neonatal period and childhood, should be performed to demonstrate the clinical utility of the prenatal Mod‐MPI evaluation.

In conclusion, this was a preliminary study suggesting that some fetuses with isolated mild‐to‐ moderate VM may have impaired cardiac function, characterized by a higher Mod‐MPI and longer IRT. This might be useful in improving fetal surveillance in isolated mild‐to‐moderate VM.
